# Effectiveness of the active communication education program in improving the general quality of life of older adults who use hearing aids: a randomized clinical trial

**DOI:** 10.1186/s12877-024-05424-0

**Published:** 2024-10-12

**Authors:** Anthony Marcotti, Sebastián Rivera, Catherine Silva-Letelier, Javier Galaz-Mella, Eduardo Fuentes-López

**Affiliations:** 1 Escuela de Fonoaudiología, Facultad de Odontología y Ciencias de la Rehabilitación, Universidad San Sebastián, Santiago, Chile; 2 Escuela de Fonoaudiología, Facultad de Salud, Universidad Santo Tomás –, Viña del Mar, Chile; 3 Programa de Magister en Epidemiología, Escuela de Salud Pública, Facultad de Medicina, Pontificia Universidad Católica de Chile, Santiago, Chile; 4 Exercise and Rehabilitation Sciences Institute, School of Speech Therapy, Faculty of Rehabilitation Sciences, Universidad Andres Bello, Santiago, 7591538 Chile; 5 Departamento de Fonoaudiología, Escuela de Ciencias de la Salud, Facultad de Medicina, Pontificia Universidad Católica de Chile, Vicuña Mackenna 4860, Macul, Región Metropolitana Chile

**Keywords:** Older adults, Hearing loss, Hearing aids, Group communication programs, Quality of life

## Abstract

**Background:**

Hearing loss in older adults affects general, generic health-related and disease-specific quality of life (QoL). The conventional strategy to address it is through hearing aids, which have been shown to improve disease-specific QoL. However, the long-term results regarding general quality of life are unknown, and communication problems and stigma associated with hearing loss may persist. An effective intervention strategy to address these problems is group communication programs, most notably Active Communication Education (ACE). This program has been shown to increase communication strategies and reduce communication activity limitations and participation restrictions. These precedents allow us to hypothesize that this program could improve general QoL.

**Methods:**

A randomized clinical trial was conducted on 114 older adult hearing aid users. Fifty-four subjects composed the intervention group that received the ACE program, while 60 subjects composed the control group that received an informational-lectures type intervention. The WHOQOL-BREF questionnaire was used to measure general QoL. Measurements were taken before and right after the intervention, with follow-ups at 6 and 12 months. Multilevel linear mixed models were estimated, considering the WHOQOL-BREF dimension scores and total score as the outcomes, and an interaction term between time since intervention and group as the predictor. Within- and between-group comparisons were made.

**Results:**

Compared to the baseline time-point, the ACE group showed significant improvements right after the intervention, and at the 6-month and 12-month follow-ups for the dimensions of psychological health, social relationships, environment, and total score. Compared to the control group, the ACE group exhibited significantly greater improvements in the social dimension at all postintervention assessments, as well as in the environment dimension and total score at the 12-month follow-up.

**Conclusions:**

The ACE program improved general QoL in terms of social relationships and environment dimensions, which lasted up to 12 months after the intervention. Therefore, ACE is positioned as an effective complement for HA users, enhancing and delivering new benefits related to broader aspects of QoL not necessarily tied to health.

**Trial registration:**

ISRCTN54021189 (retrospectively registered on 18/07/2023).

**Supplementary Information:**

The online version contains supplementary material available at 10.1186/s12877-024-05424-0.

## Background

Hearing loss (HL) is one of the most common sensory deficits in the older population [1]. HL in older adults is associated with limitations in activities of daily living [2], increased risk of falls [3], reduced physical activity [4], depression [5], cognitive impairment [6], dementia [7], and increased mortality [8]. This population presents communication disorders characterized by difficulties being understood and understanding others, mainly in noisy or reverberant environments [9].

Moreover, HL in older adults also affects quality of life (QoL) [10–13]. While there is no single definition of QoL, the World Health Organization defines it as “individuals’ perception of their position in life in the context of the culture and value systems in which they live and in relation to their goals, expectations, standards, and concerns” [14]. It is a broad and complex concept that includes physical and psychological health, but also personal beliefs, social relationships, and the relationship with the environment [14].

The impact of HL on the quality of life (QoL) of older adults has been evidenced by instruments measuring general QoL [10, 11], health-related QoL [13], and disease-specific QoL [12]. Although a distinction is not usually made, general QoL instruments evaluate all aspects impacting an individual’s life, while health-related QoL instruments focus on health aspects and reflect the impact of perceived health on an individual’s ability to live a fulfilling life [15, 16]. Health-related QoL instruments can be subdivided into generic and disease-specific. Generic instruments measure general functioning without focusing on specific health conditions, whereas disease-specific instruments directly measure the consequences of a particular disease, focusing on symptoms and functional limitations [17–19]. Disease-specific instruments designed to assess auditory aspects are termed hearing-specific [13].

The conventional strategy to address HL in older people is to use hearing aids (HAs). HAs have been associated with a lower risk of falls [20], fewer emergency visits, hospitalizations, and nights in the hospital [21], and reduced depressive symptoms [22]. When combined with a comprehensive audiological intervention, HAs might also reduce cognitive changes in older adults at higher risk of cognitive decline [23]. Additionally, HAs have been linked to improvements in hearing-specific QoL, as measured by the Hearing Handicap Inventory for the Elderly (HHIE), one of the most widely used instruments for assessing hearing-specific QoL in older adults with HL [12], with effects both in the short [24, 25] and long term [26]. These improvements are most noticeable in everyday auditory situations, such as talking with family or friends, listening to the television or radio, communicating at parties or gatherings, and conversing with others [25].

Regarding generic health-related QoL, the results of HAs use are conflicting [13]. No benefits have been observed using the SF-36 questionnaire [25], nor in the long term with EuroQol-5D (EQ-5D) [26], Short Form-12 (SF-12) or EQ-5D-5 L [27]. However, marginal improvements have been shown using the 15D instrument in the long term [28]. On the other hand, general QoL has been much less studied, with only short-term improvements reported using the WHOQOL-BREF questionnaire [29], but its long-term effects remain unknown.

The absence of long-term benefits of HAs on generic health-related QoL, can be attributed to the fact that HAs do not provide benefits in the broader aspects of QoL that these instruments assess. Additionally, HAs use do not constitute a rehabilitation process itself, which is why some users continue to experience communication problems [30, 31] and self-stigmatization associated with HL and HAs use [32]. Self-stigmatization is the process where an individual experiences negative attitudes and feelings due to possessing or believing they possess an attribute that conveys a devalued social identity [33]. In this case, the negative attribute is HL and/or the use of HAs [32]. In older adults, this process negatively impacts psychosocial aspects [34, 35] leading to reduced participation in social activities and increased loneliness and social isolation [36]. Consequently, they may adopt disengaged coping behaviors, such as avoiding or taking passive roles in social situations [34]. For these reasons, it is likely that HAs do not provide consistent long-term benefits in general QoL.

While HAs provide audibility benefits in some everyday situations, including certain social contexts, they do not guarantee the strengthening of weakened social networks or the resumption of social activities restricted by self-stigmatization [32, 34]. After 20 months of HAs fitting, older adults with restricted social networks and no social activities are less likely to experience benefits, even in hearing-specific QoL, compared to pre-fitting measurements [27]. Considering these points, it is important that rehabilitation strategies not only improve hearing-specific or health-related aspects but also enhance broader aspects of general QoL, such as social relationships, personal beliefs, and interactions with the environment.

Group communication programs are effective rehabilitation strategies for addressing both communication problems and the self-stigmatization of HL, and thus, they are positioned as a potential strategy to improve the general QoL of older adults using HAs. These interventions teach communication strategies and provide psychosocial benefits [37]. The acquisition of communication strategies constitutes a tool for engaging coping, behaviors to cope with the adverse effects of HL, such as social isolation [34], which is one of the main facilitators of social participation of older adults with HL [38]. Additionally, these interventions promote interaction with peers under the same condition, which improves the social identity devalued by the stigma of HL and increases the predisposition to participate in social activities and interactions [33].

One such program is Active Communication Education (ACE), which uses an interactive problem-solving approach [39, 40]. Participants identify their communication difficulties and underlying causes, then exchange opinions and experiences to seek solutions and learn communication strategies. This program has been shown to increase the use of communicative strategies [41, 42], and reduce communicative activity limitations and participation restrictions [39, 41, 42]. Improvements in hearing-specific QoL [39, 43, 44], auditory functioning [44], and general well-being [39] have also been reported. Despite these benefits, the ACE program has not shown effects on generic health-related QoL. Hickson et al. [39] conducted a clinical trial using the SF-36 questionnaire and found no benefits associated with ACE. Similarly, Öberg et al. [42, 45] conducted two prospective studies using the EQ-5D scale and did not find improvements in the short or long term.

To date, the effects of the ACE program on general QoL have not been studied. However, its previously reported benefits in increasing the use of communication strategies, reducing communicative activity limitations and participation restrictions, as well as potential improvements in social identity inherent in group interventions, allow us to hypothesize that the ACE program is an effective strategy for enhancing general QoL. With this in consideration, the present study aimed to evaluate the effect of the ACE program on the general QoL of older adult HAs users.

## Methods

The present study analyzed the secondary outcome of a multicenter, double-blind, randomized parallel design clinical trial (ISRCTN54021189; retrospectively registered available on https://www.isrctn.com/). The study was conducted in five Family Health Centers (CESFAMs in Spanish) distributed across two regions of Chile: two CESFAMs from San Joaquín, one from Puente Alto, and one from San Bernardo, all communes of the Metropolitan Region, and one CESFAM located in the commune of Valparaíso, in the Valparaíso Region. These centers are public primary health care facilities that operate under a family and community approach and are geographically located close to the homes of the people they cover. The current study did not consider adverse effects or risks, whether direct or indirect. No modifications were made to the methodology following the commencement of the trial. The trial concluded when all follow-ups were completed.

### Participants

**Fig. 1:**
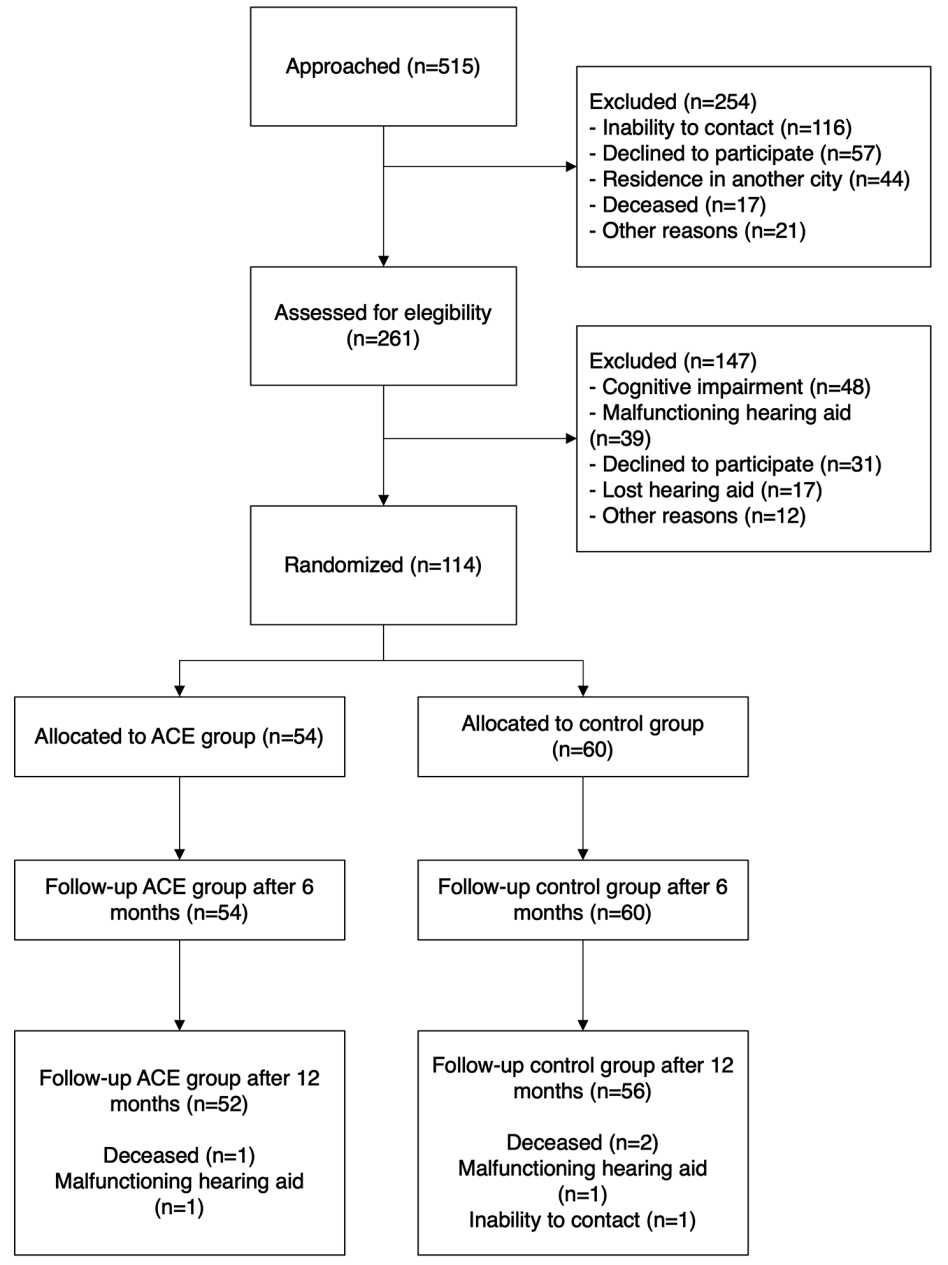
CONSORT study flowchart by treatment strategy. ACE: Active Communication Education

The participants were older adults (≥ 65 years) who had been fitted with HAs in at least one ear at any time in the last five years through Chile’s public health system (within this system, HAs are provided at no cost or with a nominal co-payment not exceeding 20% of their price) [46], without cognitive impairment according to the Chilean version of the Mini-Mental State Examination (MMSE) (≥ 22 score) [47]. Patients with technical issues or lost HAs were excluded. All patients were recruited between October 2019 and October 2020. All participants signed an informed consent prior to their participation. No incentives were offered to the participants. The flow diagram of the study participants is shown in Fig. 1. The study sample can be considered sociodemographically representative of older adults in Chile since 92% of this population is served by the public health system (Fondo Nacional de Salud - FONASA) [48]. Additionally, all participants belonged to categories A and B of FONASA, which determine the copayment level based on beneficiaries’ income. These categories include 80% of people over 65 years old, whose monthly incomes are below $440,000 Chilean pesos (approximately $480 USD) [48].

### Randomization

Participants were randomized utilizing computer-generated stratified blocks by region, with blocks of six participants used to create four possible assignment combinations. The allocation of patients to control or treatment groups within blocks is done in a way that ensures randomization while maintaining the desired allocation ratios within each block [49]. The control group comprised 60 participants, with 36 from the Metropolitan Region and 24 from the Valparaíso Region. The intervention group consisted of 54 participants, including 34 from the Metropolitan Region and 20 from the Valparaíso Region.

### Blinding

It is recommended that, whenever possible, blinding should be used in randomized clinical trials. Blinding of at least the trial participants and treatment providers helps to reduce information bias [50]. In psychosocial interventions, it is recommended to incorporate all possible blinding procedures [51]. In line with these recommendations, a designated team member maintained the information necessary to preserve the intended blinding. Participants were informed of both existent groups but did not know their assigned group until after participating. Therapists were unaware of which program they would be applying, only that they would be applying a new type of group intervention. Additionally, the data collection personnel were kept unaware; subjects were randomly assigned by the designated team member, enabling the evaluators to manage both groups without bias.

### Interventions

The interventions started two months after recruitment, with 6- and 12-months follow-ups after the intervention. The programs were carried out by four trained speech and language therapists: two in the ACE program and two in an informational lecture-type program. In Chile, speech and language therapists have specialized training in audiology, so they are the professionals in charge of providing assessment and rehabilitation services in the field of hearing. Before starting the interventions, the professionals received a briefing on the respective programs to be used in the study. In this briefing, they were informed about their roles in each program, the duration and frequency of the sessions, the topics to be covered each week, and the expected group dynamics.

The ACE group, composed of 9 subgroups of between 5 and 6 people, received the Chilean adaptation of the ACE program [52]. This version consists of a weekly 90-minute group session for six weeks. Through dynamic group sessions, participants must identify their daily communication difficulties and possible strategies to solve them, practice them, decide which ones suit their needs, and transfer them to their routines. The professional in charge of the group takes on the role of facilitator and moderator of the conversations among the participants.

In the first session, the rules and structure of the program were presented, experiences with HL were shared, and introductory topics such as hearing in old age and the Chilean national public policy on HAs were addressed. In the second session, communication needs, problems, and strategies to solve them were identified and discussed. The third session discussed different types of communication strategies and their application in everyday situations. The fourth session focused on understanding speech in noisy environments and how to overcome these challenges. In the fifth session, communication with difficult speakers was addressed. The sixth session focused on difficulties in hearing other sounds, such as telephones and doorbells, the usefulness of lip reading and strategies for recognizing visemes and other facial movements associated with certain sounds.

The control group, composed of 10 subgroups of 5 to 6 people, participated in an informational lecture-type intervention, which was designed especially for this study. The professionals in charge delivered information related to aging and hearing loss through an expository format. The topics covered in each session were selected to interest the group members and maintain their attendance. This intervention had the same frequency and duration as the ACE and did not include social interaction activities among participants.

In the first session, the functioning of HAs and national public policies targeting individuals with HL were explained. The second session focused on age-related HL and rehabilitation options. The third session presented the biological changes associated with aging and which specialist to consult in case of difficulties. The fourth session addressed the cognitive changes and difficulties of aging and their impact on communication. In the fifth session, participants received guidance on hearing self-care related to noise exposure and other habits. Finally, the sixth session summarized the topics of previous sessions and provided guidance on when to consult a hearing care professional.

### Outcomes

The data were collected before the intervention, right after the intervention, and at the 6- and 12-months follow-ups after the intervention. The outcome of this study was the general QoL measured with the WHOQOL-BREF questionnaire [53], which was previously validated in older Chilean adults [54]. This instrument assesses QoL as a multidimensional construct composed of four dimensions: physical health, psychological health, social relationships, and environment (Table S1). This instrument has two general items and 24 items that assess each dimension. Each item has five possible Likert-type responses (not at all/very bad/very dissatisfied: 1 point; little/regular/poorly satisfied: 2 points; moderate/average: 3 points; somewhat/very good/reasonably satisfied: 4 points; very good/extremely/very satisfied: 5 points). A scoring scale of 4 to 20 points per dimension was used, with a total score of 80 points [53], where the higher the score is, the greater the quality of life.

### Statistical analysis

Multilevel linear mixed models were estimated, considering the WHOQOL-BREF dimension score and total score as the outcomes. The predictor was an interaction term between time since intervention and group. The models were adjusted for the respective estimates of baseline scores to correct for significant baseline differences observed in an unadjusted model (Table S2). Estimations were performed with random intercept (within-subject), robust variance, and bootstrapping (1000 repetitions). Marginalized predictions were used for each group at each time point to obtain the estimated scores. Comparisons between and within groups were made by linear combinations based on these estimates, using the baseline as reference. Finally, effect sizes of between-group differences were estimated using Cohen’s *d*, which were interpreted according to Sawilowsky’s suggestions [55]. All analyses were performed with STATA v18 software, and the figures were generated with the “ggplot2” library of RStudio software.

## Results

### Sample description

Contact information was obtained for 515 hearing aid users seen at the participating primary health care centers. Of these, 261 were screened for eligibility. Finally, 114 patients met the inclusion/exclusion criteria and agreed to participate in the study. The ACE group comprised 54 subjects, and the control group comprised 60 (details in Fig. 1). The sample had a median age of 79 years, an average of 6 years of education, and 66 (57.89%) women. No significant differences were observed between groups for any sociodemographic or clinical variable (Table 1). The observed WHOQOL-BREF scores can be found in Table S3.

**Table 1:** Characteristics of the sample of patients involved in the control group and ACE group (*n* = 114)

Variable	Control group	ACE group	p-value^a^
	x̄ (SD) o p50 (p25-p75)	x̄ (SD) o p50 (p25-p75)	
**Age**	79 (75.5–85.0)	79 (72.0–85.0)	0.498
**Education**	6 (3.5–10.0)	7 (6.0–12.0)	0.056
**Number of women**	37 (61.67%)	29 (53.70%)	0.390
**Self-perception of state of health**			
Excellent	2 (3.33%)	3 (5.56%)	0.617
Very good	2 (3.33%)	3 (5.56%)	
Good	21 (35.0%)	16 (29.63%)	
Alright	28 (46.67%)	21 (38.89%)	
Bad	7 (11.67%)	11 (20.37%)	
**Number of chronic illnesses**	2 (1–3)	2 (1–3)	0.606
**Monthly income (in chilean pesos)**	180,000 (150,000-230,000)	180,000 (130,000-220,000)	0.912
**Monthly spending on medication (in chilean pesos)**	125,000 (0,00-300,000)	150,000 (0,00-300,000)	0.562
**How many people do they live with?**			
1	10 (16.67%)	7 (12.96%)	0.708
2	24 (40.00%)	25 (46.30%)	
3	13 (21.67%)	9 (16.67%)	
4	12 (20.00%)	10 (18.52%)	
5	1 (1.67%)	3 (5.56%)	
**MMSE**	25 (23.0-27.5)	27 (23.0–28.0)	0.317
**(Yesavage) Depression Scale**	4 (2–6)	4 (2–7)	0.306
**Willingness to use hearing aid**	3 (1.5-5)	3 (1–4)	0.247
**Pure Tone Average (PTA)**	60.30 (50.60-68.45)	63.55 (52.50–74.40)	0.179
**Years of experience with hearing aids**			
Left ear	0 (0-2.50)	0 (0-1.8)	0.605
Right ear	1 (0–3)	1 (0-3.08)	0.962

^a^ To assess the success of randomization, the chi2 test was used to compare categorical variables and Mann-Whitney´s non-parametric test to compare continuous variables

MMSE = Mini-Mental State Examination

### Within group comparison

**Fig. 2:**
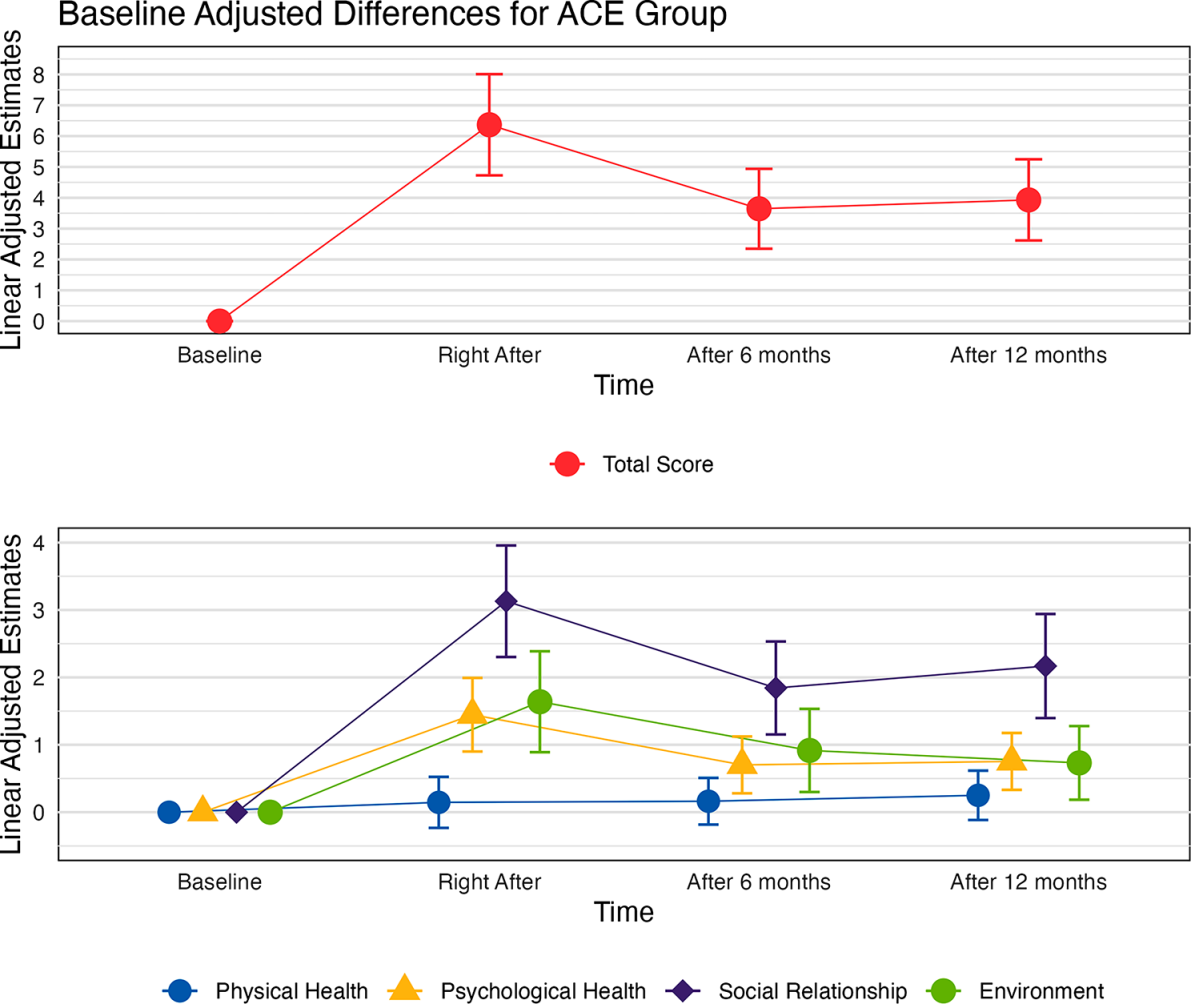
Baseline adjusted differences in total score and each of the dimensions in relation to the baseline time-point for the WHOQOL-BREF questionnaire for the ACE group

The effect of the intervention over time was modeled for each of the dimensions and the total score (Table S4). Compared to the baseline timepoint, in the ACE group, significant increases in psychological health (Δβ = 1.45; *p* < 0.001), social relationships (Δβ = 3.13; *p* < 0.001), environment (Δβ = 1.64; *p* < 0.001) and the total score (Δβ = 6.37; *p* < 0.001) were observed right after intervention. The same was observed after 6 months for psychological health (Δβ = 0.70; *p* < 0.05), social relationships (Δβ = 1.84; *p* < 0.001), environment (Δβ = 0.92; *p* < 0.05) and total score (Δβ = 3.64; *p* < 0.001) and after 12 months for psychological health (Δβ = 0.75; *p* < 0.001), social relationships (Δβ = 2.17; *p* < 0.001), environment (Δβ = 0.73; *p* < 0.05) and total score (Δβ = 3.93; *p* < 0.001) (Fig. 2).

**Fig. 3:**
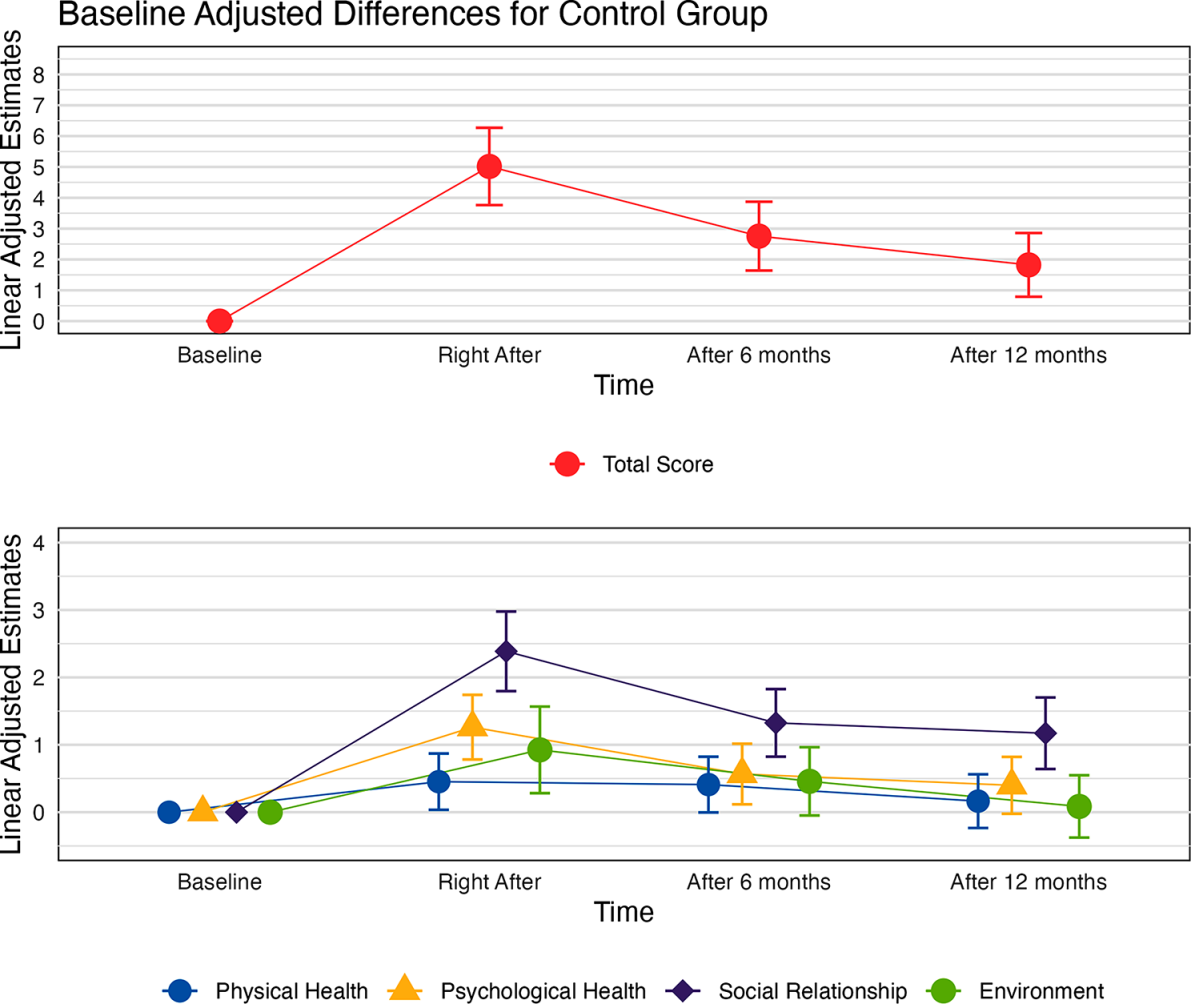
Baseline adjusted differences in total score and each of the dimensions in relation to the baseline time-point for the WHOQOL-BREF questionnaire for the control group

In the control group, significant increases in physical health (Δβ = 0.45; *p* < 0.05), psychological health (Δβ = 1.26; *p* < 0.001), social relationships (Δβ = 2.39; *p* < 0.001), environment (Δβ = 0.93; *p* < 0.05) and the total score (Δβ = 5.02; *p* < 0.001) were observed right after intervention. The same occurred after 6 months for psychological health (Δβ = 0.57; *p* < 0.05), social relationships (Δβ = 1.33; *p* < 0.001) and total score (Δβ = 2.76; *p* < 0.001), and after 12 months for social relationships (Δβ = 1.17; *p* < 0.001) and total score (Δβ = 1.82 *p* < 0.05) (Fig. 3).

### Between groups comparison

No significant differences were observed between groups at any of the time points, except for social relationships in favor of the ACE group right after intervention (*p* < 0.05), after 6 months (*p* < 0.05) and after 12 months (*p* < 0.05), as well as in environment (*p* < 0.05) and total score (*p* < 0.001) after 12 months (Table 2). In addition, after 12 months, a moderate effect size was observed for total score (*d* = 0.70), social relationships (*d* = 0.65), environment (*d* = 0.60), and a small effect was observed for physical health (*d* = 0.01) and psychological health (*d* = 0.17).

**Table 2:** Between groups differences (ACE group - control group) on linear estimates for the WHOQOL-BREF questionnaire and its dimensions

	Right after		After 6 months		After 12 months	
	Δβ (CI 95%)		Δβ (CI 95%)		Δβ (CI 95%)	
	**Between groups**	**p-value**	**Between groups**	**p-value**	**Between groups**	**p-value**
Total Score^**a**^	1.05 (-0.38 to 2.48)	0.149	0.59 (-0.38 to 1.55)	0.232	1.81 (0.84 to 2.78)	**< 0.001**
Physical Health^**a**^	-0.39 (-0.80 to 0.03)	0.067	-0.33 (-0.70 to 0.04)	0.082	0.01 (-0.38 to 0.39)	0.974
Psychological Health^**a**^	-0.00 (-0.52 to 0.52)	0.994	-0.05 (-0.43 to 0.33)	0.789	0.17 (-0.21 to 0.55)	0.384
Social Relationships^**a**^	0.79 (0.11 to 1.47)	**0.023**	0.57 (0.06 to 1.07)	**0.029**	1.05 (0.45 to 1.64)	**0.001**
Environment ^**a**^	0.72 (-0.03 to 1.46)	0.060	0.46 (-0.04 to 0.96)	0.071	0.65 (0.26 to 1.04)	**0.001**

Statistically significant values are highlighted in black

^**a**^ Baseline-adjusted models

## Discussion

### Improvements in the ACE group

The present study aimed to evaluate the effect of the ACE program on general QoL in older adult users of HAs. To date, this is the first study to do so. This program showed significant improvements right after, 6 months after, and 12 months after the intervention in relation to the baseline, in the dimensions of psychological health, social relationships, and environment, in addition to the total score.

These improvements can be attributed to the communicative strategies addressed by ACE and to the psychosocial benefits of interacting with peers. On the one hand, using communicative strategies is part of engaging coping behaviors, one of the main facilitators of social participation in older adults with HL [38]. Social participation, in turn, is associated with better general QoL [56]. On the other hand, interaction with peers may provide psychosocial benefits in normalizing the self-stigma of HL. This interaction allows people to share their difficulties and experiences, feeling less isolated and more understood [33]. With this, they regain a positive social identity and improve their willingness to participate in social activities and interactions [33], which could also influence general QoL.

Although the ACE program and a general QoL instrument were not used, the clinical trial by Preminger & Yoo [57] showed similar results for two different group interventions. These authors compared the effects of one intervention involving communicative strategies training, one involving communicative strategies training plus psychosocial strategies, and the third involving informational lectures plus psychosocial strategies on health related QoL. The generic health-related QoL was measured using the World Health Organization Disability Assessment Schedule II (WHODAS II). At 6 months postintervention, the groups whose interventions incorporated psychosocial strategies improved significantly on two subscales of the WHODAS II: Getting Along With People (establishing and maintaining relationships with significant others) and Participation in Society (barriers to participation in society due to health difficulties).

However, other studies have not obtained the same results using generic health-related QoL instruments. Hickson et al. [39] conducted a clinical trial in which they studied the effects of ACE on the generic health-related QoL of older adult users and nonusers of HAs. They compared a group that received the ACE program with a control group that sequentially received a placebo social program and then ACE. After receiving the intervention via the ACE program, neither group showed significant improvements in the health-related QoL measured with the SF-36. However, there was evidence of a reduction in participation restrictions (Quantified Denver Scale of Communicative Function - QDS) and communicative activity limitations (Self-Assessment of Communication - SAC) in both groups, which were maintained 6 months after the intervention.

In a prospective study, Öberg et al. [45] also evaluated the effect of ACE on older adult users and nonusers of HAs. They evaluated the health-related QoL with the EQ-5D questionnaire and found no improvement at 3 weeks or 6 months postintervention. In a similar study, Oberg et al. [42] obtained the same results using only the EQ-5D visual analog scale 6 months postintervention. However, in this last report, the authors did show an increase in the use of communication strategies (Communication Strategies Scale - CSS) and an improvement in hearing-specific QoL (HHIE), results that have been corroborated in a subsequent study [41].

The differences between our study and those of Hickson et al. [39] and Öberg et al. [42, 45] can be attributed mainly to the differences in the instruments used. The SF-36 and EQ-5D are measures of generic health-related QoL focused on functioning and perceptions related exclusively to health status [58, 59]. In contrast, the WHOQOL-BREF measures general QoL based on a broader definition [60] that incorporates individual perceptions of health status, but also more general aspects of life, such as personal beliefs, social relationships, and the relationship with the environment [14]. Although they have similarities, these instruments measure different QoL constructs both theoretically [15, 16, 58] and empirically [60]. For this reason, the WHOQOL-BREF could be a more sensitive instrument for revealing improvements in psychosocial aspects, such as those provided by the ACE program. This would also explain why, despite having evidenced this type of benefits, the studies by Hickson et al. [39] and Öberg et al. [42, 45] did not show improvements in generic health-related QoL.

Individual differences among study participants also could explain these contrasting results. Hickson et al. [39] and Öberg et al. [45] included individuals who did not use HAs in their research. Self-reported activity limitations or participation restrictions are determining factors for seeking help with HL and opting for HAs [61]. Therefore, these subjects are expected to have experienced fewer difficulties in these aspects than peers with HAs. This implies that they probably did not experience significant impacts on their generic health-related QoL, thus minimizing the chances of observing improvements. The same could have happened with the second study by Oberg et al. [42], in which subjects with severe difficulties communicating in groups were excluded.

Furthermore, Hickson et al. [39] included some individuals with HL with normal hearing in the better ear and, in the study by Öberg et al. [45], the vast majority had mild and moderate HL in the better ear. It has been shown that the greater the severity of HL is, the greater the impact on the generic health-related QoL [30]. For this reason, subjects with milder HL may not have significantly impacted their generic heath-related QoL, leaving a reduced or no margin to experience improvements with ACE. In the present study, all subjects were implemented with HAs through the Chilean public health system, one of whose requirements is to have bilateral HL of moderate or greater degree in the better ear [46]. This suggests that participants in this study experienced a greater impact on QoL (both generic health-related and general) at baseline than participants in other studies and were, therefore, more likely to benefit from ACE.

### Improvements in the control group

A finding not hypothesized in the present study was improvements in the control group. Although most of these improvements tended to diminish at 6 months, they were maintained at 12 months. Similarly, the control group of Hickson et al. study [39] that received a placebo social intervention had a reduction in participation restrictions (QDS) and an improvement in the mental health dimension of the SF-36. After the same group of patients were treated with the ACE, they showed improvements in their perceived general well-being (Ryff Psychological Well-Being Scale) which lasted for 6 months. This did not occur for the group that only received the ACE program.

Rivera et al. [44] reported similar results using a hearing-specific instrument (HHIE). Through an exploratory prospective cohort study, these authors compared a group that received the ACE program with a control group that received a cognitive stimulation program. Both groups improved immediately after the intervention. When comparing these improvements, no significant differences between groups were evident.

In the present study, the control group may have experienced some unplanned psychosocial benefits from peer interaction. Although the control group intervention involved informational lectures and did not include social interaction activities, it is to be expected that participants would interact while waiting for the activity to begin or at the end of each session. They may have shared experiences or opinions during the development of the sessions. It has been described that unstructured psychosocial exercises also provide benefits [62] and that social interactions without therapeutic objectives improve general QoL as measured by the WHOQOL-BREF [63].

### Effectiveness of ACE

Compared to the control group, improvements in the social dimension in the ACE group were significantly greater at all postintervention measurements. In addition, the improvements obtained in the environmental dimension were significantly greater at the 12-month measurement. These differences had a moderate effect size. These results can be attributed to the specific characteristics of ACE. Through communicative strategies, this program seeks to reduce communicative activity limitations and participation restrictions [40]. ACE has already been shown improvements in these aspects [39] and increase the use of communicative strategies [41, 42]. In addition to interactions with peers, these characteristics would improve subjects’ perception of the quantity and quality of their social relationships and interactions.

On the other hand, the communicative strategies of the ACE program also improve functional aspects of hearing in everyday situations [44]. These strategies include understanding others in public places, such as stores, transportation, and streets. This would provide a sense of control and security for conversations outside the home, resulting in a more positive perception of the environment and the immediate surroundings.

Both the intervention of the control group and the ACE group had the same frequency and duration. For this reason, the significantly greater benefits obtained by the ACE group can be attributed to both the content and the interactive problem-solving approach inherent to the ACE program [39, 40]. Furthermore, the Chilean version of the ACE was previously adjusted to the identified needs of the target population [44]. This means that the duration, modality, and content of the program were tailored to the strengths and weaknesses identified in this population, both by the older adults and by the professionals implementing the program. For this reason, the Chilean version of the ACE program is designed to be effective in its entirety; thus, reducing its duration or content, or modifying its group dynamics, would likely not yield the same results. These facts also suggest that an unstructured psychosocial dynamic would not be sufficient to achieve the same effects.

Finally, it is important to mention that the ACE program is a cost-effective, easy-to-implement rehabilitation strategy capable of reaching multiple users simultaneously. Detailed manuals are available in English, Spanish, and German on the official ACE website (https://shrs.uq.edu.au/active-communication-education-ace). For this reason, ACE can be uniformly implemented in various settings, such as meeting rooms in public libraries, retirement villages, church halls [39], community centers [42], or primary health care centers [44]. In our study, the ACE program was implemented in CESFAMs, a type of public primary health care facility located close to the homes of the people they serve. The versatility and benefits of the ACE program make it an effective alternative for public policies providing HAs or rehabilitation for older adults with HL, offering accessible, community-based care.

### Limitations

One of the main limitations of this study was the absence of measurements of communicative activity limitations, participation restrictions, use of communicative strategies, and engaged coping behaviors due to the absence of instruments in Spanish. As mentioned, the ACE program has been shown to improve some of these aspects [39, 41, 42], possibly serving as intermediate variables between the ACE and the general QoL. Future research should consider these elements to further study the mechanisms of action of this and other communicative programs on general QoL.

A second limitation is that we did not quantify the degree of social interaction experienced by the participants in their respective interventions. The observed improvements in the control group can be attributed to psychosocial benefits from interaction with peers with HL [33]. Considering this variable in further studies would allow individualizing the benefits provided by the specific characteristics of the communicative programs from those attributable to peer interaction.

## Conclusion

The ACE program proved to be an effective intervention for improving general QoL in older adults with HL who are users of HAs. Specifically, improvements occurred in the dimensions of social relationships and perception of the environment and immediate surroundings, which persisted up to 12 months after the intervention. These benefits can be attributed to the communicative strategies addressed by the program and the interaction with peers under the same condition. For these reasons, ACE is positioned as an effective complement for HAs users, enhancing and delivering new benefits related to broader aspects of QoL not necessarily tied to health.

## Electronic supplementary material

Below is the link to the electronic supplementary material.

Supplementary Material 1

## Data Availability

The dataset used and analyzed during the current study is available from the corresponding author upon reasonable request.

## Abbreviations

HL: Hearing Loss
QoL: Quality of Life
HAs: Hearing Aids
CESFAMs: Centro de Salud Familiar (Family Health Centers)
MMSE: Mini-Mental State Examination
EQ-5D: EuroQol-5D
SF-36: Short Form-36
SF-12: Short Form-12
ACE: Active Communication Education
CSS: Communication Strategies Scale
QDS: Quantified Denver Scale of Communicative Function
SAC: Self-Assessment of Communication
WHODAS II: World Health Organization Disability Assessment Schedule II
WHOQOL-BREF: World Health Organization Quality of Life Instrument, Brief Version
