# Supplementary material for: Effectiveness of the active communication education program in improving the general quality of life of older adults who use hearing aids: a randomized clinical trial
Source: BMC Geriatr. 2024 Oct 12;24:828. doi: 10.1186/s12877-024-05424-0 (PMC11470628; doi:10.1186/s12877-024-05424-0)
Supplement: Supplementary file 1 — Supplementary Material 1 [file 12877_2024_5424_MOESM1_ESM.docx]

**Table Supplementary 1**

| **Table S1.** *Description of the dimensions and their facets of the WHOQOL-BREF questionnaire* | | |
| --- | --- | --- |
| **Dimension** | **Facet** | **Description** |
| **Physical health** | Pain and discomfort | Unpleasant physical sensations such as long-term or short-term pain, stiffness, aches and itches, experienced by a person and, the extent to which these are distressing and interfere with life. |
|  | Energy and fatigue | Explores energy, enthusiasm, endurance, and disabling tiredness regarding perform various daily tasks |
|  | Sleep and rest | How much sleep, rest as well as issues with initiating, maintaining, or waking up at inappropriate hours, impact the quality of life |
| **Psychological health** | Positive feelings | How much a person experiences positive feelings of contentment, balance, peace, happiness, hopefulness, joy and enjoyment of the good things in life, as well as feelings about the future. |
|  | Thinking, learning, memory, and concentration | Explores thinking, learning, memory, concentration and ability to make decisions, in regard to speed of thinking and clarity of thought |
|  | Self-esteem | Explores how a person feel about themselves in terms of sense of worth, self-efficacy, satisfaction with oneself and control. |
|  | Body image and appearance | Explores the persons view of their body in a positive or negative way, and the effect on their self-concept |
|  | Negative feelings | Explores how much a person experiences negative feelings such as despondency, guilt, sadness, tearfulness, despair, nervousness, anxiety, and lack of pleasure in life |
| **Social relationships** | Personal relationships | Examines the extent to which people cares and provides for others, feel the companionship, love and support they desire from the relationships in their life. Addresses all kinds of relationships such as friendships and marriages. |
|  | Social support | Explores how much a person feels the commitment, approval, encouragement, and availability of practical assistance from family and friends, in regard to solver personal and family problems. |
|  | Sexual activity | Explores a person's sex drive, sexual expression and sexual fulfilment, and the extent to be able to express and enjoy their sexual desire. |
| **Environment** | Physical safety and security | Explores the person's sense of safety, freedom, and of security from physical harm. |
|  | Home environment | Explores the principal place where a person lives, regarding being comfortable, safe, crowded, space available, cleanliness, privacy, facilities available, and quality of construction of the building. Immediate neighborhood is also explored. |
|  | Financial resources | Examines the person's view of how their financial resources and the extent to which these resources allow the person to afford their needs, which might affect their quality of life |
|  | Health and social care: availability and quality | How the person views the availability, quality and completeness of the health and social services as well as how easy/difficult is to reach local health and social services |
|  | Opportunities for acquiring new information and skills | Explores a person's opportunity and desire to learn new skills, acquire new knowledge, and feel in touch with what is going on, through formal education programs, education classes, or recreational activities, either in groups or alone. Includes being in touch and having new of what is going on. |
|  | Participation in and opportunities for recreation and leisure | Explores a person's ability, opportunities, inclination, and enjoyment to participate in leisure, pastimes, relaxation, and recreation. |
|  | Physical environment (pollution/noise/  traffic/climate) | Explores the person's view of his/her environment regarding noise, pollution, climate and aesthetics of the environment, and how this serves to improve or adversely affect quality of life. |
|  | Transport | Explores the person's view of how available it is to find and use transport services to get around, to perform the tasks of daily life as well as to perform chosen activities |

**Table Supplementary 2**

Upon examining the linear estimates, there were no significant differences between groups in terms of overall baseline score. However, for the physical and psychological dimensions, a significant difference was observed at this time point (Table Supplementary 2). When considering the magnitude of this differences, the value remains relatively constant across subsequent time points, indicating that the initial difference was sustained over time. Thus, the observed differences over time seem to be associated with this initial difference maintained in later measurements.

| **Table S2.** | | | | | | | | | | | | |
| --- | --- | --- | --- | --- | --- | --- | --- | --- | --- | --- | --- | --- |
| *Linear estimates for the WHOQOL-BREF questionnaire and its dimensions* | | | | | | | | | | | | |
|  | **Baseline** | | | **Right after** | | | **After 6 months** | | | **After 12 months** | | |
|  | **B (CI 95%)** | | | **B (CI 95%)** | | | **B (CI 95%)** | | | **B (CI 95%)** | | |
|  | **ACE** | **Control** | **p-value** | **ACE** | **Control** | **p-value** | **ACE** | **Control** | **p-value** | **ACE** | **Control** | **p-value** |
| Total Score | 51.57 (50.22-52.91) | 52.88 (51.85-53.91) | 0.133 | 57.93 (56.81-59.06) | 57.90 (57.01-58.78) | 0.957 | 55.21 (54.47-55.95) | 55.64 (54.90-56.37) | 0.417 | 55.51 (54.74-56.27) | 54.68 (53.96-55.40) | 0.134 |
| Physical Health | 12.75 (12.45-13.05) | 13.27 (12.91-13.63) | **0.035** | 12.90 (12.58-13.22) | 13.72 (13.43-14.01) | **<0.001** | 12.91 (12.65-13.18) | 13.68 (13.40-13.95) | **<0.001** | 13.00 (12.69-13.31) | 13.43 (13.15-13.70) | **0.046** |
| Psychological Health | 13.45 (13.02-13.87) | 14.38 (13.99-14.78) | **0.002** | 14.89 (14.50-15.28) | 15.65 (15.31-15.98) | **0.004** | 14.15 (13.91-14.39) | 14.95 (14.68-15.23) | **<0.001** | 14.18 (13.91-14.46) | 14.78 (14.49-15.06) | **0.004** |
| Social Relationships | 12.16 (11.47-12.85) | 12.00 (11.53-12.46) | 0.710 | 15.29  (14.77-15.80) | 14.38 (13.98-14.79) | **0.007** | 14.00 (13.59-14.41) | 13.32 (12.98-13.67) | **0.014** | 14.33 (13.87-14.79) | 13.16 (12.79-13.53) | **<0.001** |
| Environment | 13.23 (12.67-13.79) | 13.23 (12.77-13.68) | 0.986 | 14.87  (14.35-15.39) | 14.15  (13.65-14.65) | 0.051 | 14.15 (13.80-14.50) | 13.68 (13.35-14.02) | 0.063 | 13.97 (13.67-14.28) | 13.32 (13.02-13.61) | **0.003** |
|  | | | | | | | | | | | | |

**Table Supplementary 3**

| **Table S3.** | | | | | | | | | | | | | | | | | | |
| --- | --- | --- | --- | --- | --- | --- | --- | --- | --- | --- | --- | --- | --- | --- | --- | --- | --- | --- |
| *Observed scores for the WHOQOL-BREF questionnaire and each of its dimensions.* | | | | | | | | | | | | | | | | | | |
|  | **Baseline**  **p50 (p25-p75)** | | | | **Right After**  **p50 (p25-p75)** | | | | | **After 6 months**  **p50 (p25-p75)** | | | | | **After 12 months**  **p50 (p25-p75)** | | | |
|  | **ACE** | **Control** | **p-value** |  | | **ACE** | **Control** | **p-value** |  | | **ACE** | **Control** | **p-value** |  | | **ACE** | **Control** | **p-value** |
| Total Score | 51.50  (42.8-60.00) | 52.45 (47.90-56.25) | 0.721 |  | | 58.95 (51.20-65.50) | 57.35 (54.25-61.85) | 0.715 |  | | 55.65 (48.90-60.50) | 55.00 (51.60-58.30) | 0.882 |  | | 56.10 (48.25-61.00) | 54.35 (51.35-58.31) | 0.751 |
| Physical Health | 12.60 (10.90-14.90) | 13.01 (11.40-15.40) | 0.280 |  | | 12.60 (10.90-15.40) | 13.70 (12.00-15.40) | 0.079 |  | | 12.60 (10.90-15.40) | 13.70 (12.00-15.70) | 0.062 |  | | 12.85 (10.90-15.15) | 13.10 (12.00-15.40) | 0.220 |
| Psychological Health | 13.65 (12.00-16.00) | 14.00 (12.35-16.00) | 0.253 |  | | 15.30 (12.70-17.30) | 16.00 (14.00-17.30) | 0.348 |  | | 14.70 (12.70-16.70) | 15.30 (13.30-16.70) | 0.160 |  | | 14.70 (12.70-16.70) | 15.30 (13.30-16.70) | 0.208 |
| Social Relationships | 12.00 (9.30-14.70) | 12.00 (9.30-14.00) | 0.677 |  | | 16.00 (13.30-17.30) | 14.70 (12.00-16.00) | **0.017** |  | | 14.70 (12.00-16.00) | 13.30 5(11.00-14.70) | 0.151 |  | | 14.70 (12.65-16.00) | 13.30 (10.70-14.70) | 0.054 |
| Environment | 13.75 (11.00-15.50) | 13.00 (11.75-14.25) | 0.840 |  | | 15.25 (13.00-17.00) | 13.75 (12.25-17.00) | 0.091 |  | | 14.25 (12.00-15.50) | 13.25 (12.00-15.50) | 0.259 |  | | 14.00 (11.50-15.50) | 13.00 (11.50-15.00) | 0.231 |
|  | | | | | | | | | | | | | | | | | | |

No statistically significant differences were observed between the groups regarding the scores obtained for the questionnaire on the baseline time point (Table S1). However, immediately after the intervention, a significant difference was observed in social relationships in favor of the ACE group (p<0.05).

**Table Supplementary 4**

In Table S5, the multivariate mixed-effects linear model adjusted for the baseline is presented for the estimation of the total score and each of the dimensions of the WHOQOL-BREF questionnaire.

| **Table S4.** *Multivariate mixed-effects linear models for the WHOQOL-BREF questionnaire and each of its dimensions* | | | | | | | | | | |  |
| --- | --- | --- | --- | --- | --- | --- | --- | --- | --- | --- | --- |
|  | **Total Score^a^** | | **Physical Health^a^** | | **Psychological Health^a^** | | **Social Relationships^a^** | | **Environment** | | |
|  | **β (CI 95%)** | **p-value** | **β (CI 95%)** | **p-value** | **β (CI 95%)** | **p-value** | **β (CI 95%)** | **p-value** | **β (CI 95%)** | **p-value** | |
| **Time since intervention** |  |  |  |  |  |  |  |  |  |  | |
| Baseline | *Reference* | *-* | *Reference* | *-* | *Reference* | *-* | *Reference* | *-* | *Reference* | *-* | |
| Right After | 5.02 (3.76 to 6.27) | **<0.001** | 0.45 (0.04 to 0.87) | **0.033** | 1.26 (0.78 to 1.74) | **<0.001** | 2.39 (1.80 to 2.98) | **<0.001** | 0.93 (0.28 to 1.57) | **0.005** | |
| After 6 months | 2.76 (1.64 to 3.87) | **<0.001** | 0.41 (-0.00 to 0.82) | 0.052 | 0.57 (0.12 to 1.02) | **0.013** | 1.33 (0.82 to 1.83) | **<0.001** | 0.46 (-0.05 to 0.96) | 0.076 | |
| After 12 months | 1.82 (0.79 to 2.86) | **0.001** | 0.16 (-0.23 to 0.56) | 0.419 | 0.40 (-0.02 to 0.82) | 0.064 | 1.17 (0.64 to 1.70) | **<0.001** | 0.09 (-0.38 to 0.55) | 0.715 | |
| **Intervention group** |  |  |  |  |  |  |  |  |  |  | |
| Control group | *Reference* | *-* | *Reference* | *-* | *Reference* | *-* | *Reference* | *-* | *Reference* | *-* | |
| ACE group | -0.30 (-1.65 to 1.05) | 0.662 | -0.08 (-0.45 to 0.29) | 0.674 | -0.19 (-0.67 to 0.29) | 0.446 | 0.05 (-0.65 to 0.75) | 0.893 | 0.00 (-0.60 to 0.60) | 0.995 | |
| **Interaction between time and group** |  |  |  |  |  |  |  |  |  |  | |
| ACE and baseline | *Reference* | *-* | *Reference* | *-* | *Reference* | *-* | *Reference* | *-* | *Reference* | *-* | |
| ACE and right after | 1.35 (-0.72 to 3.43) | 0.201 | -0.31 (-0.87 to 0.26) | 0.288 | 0.19 (-0.55 to 0.92) | 0.623 | 0.74 (-0.28 to 1.76) | 0.153 | 0.71 (-0.28 to 1.71) | 0.159 | |
| ACE and after 6 months | 0.89 (0.82 to 2.60) | 0.308 | -0.25 (-0.79 to 0.30) | 0.374 | 0.14 (-0.49 to 0.76) | 0.673 | 0.52 (-0.35 to 1.38) | 0.239 | 0.46 (-0.34 to 1.26) | 0.261 | |
| ACE and after 12 months | 2.11 (0.40 to 3.82) | **0.016** | 0.09 (-0.46 to 0.63) | 0.757 | 0.36 (-0.26 to 0.97) | 0.256 | 1.00 (0.04 to 1.96) | **0.041** | 0.65 (-0.06 to 1.35) | 0.073 | |
| Statistically significant values are highlighted in black  **^a^** Baseline-adjusted models | | | | | | | | | | |  |
